# Rethinking Common Diagnoses: Idiopathic Multicentric Castleman Disease Presenting as TAFRO Syndrome: A Case Report

**DOI:** 10.1002/ccr3.73663

**Published:** 2026-09-30

**Authors:** Divita Rohatgi, Annesha Chakraborty, Sourav Kumar, Anurag Rohatgi

**Affiliations:** ^1^ Internal Medicine Hamdard Institute of Medical Sciences and Research Delhi India; ^2^ Internal Medicine Maulana Azad Medical College Delhi India

**Keywords:** castleman disease, idiopathic multicentric Castleman disease, lymph node biopsy, lymphadenopathy, TAFRO syndrome

## Abstract

Idiopathic multicentric Castleman disease (TAFRO subtype) can mimic common differentials. High suspicion is required for chronic systemic symptoms and generalized lymphadenopathy. As fine‐needle aspiration is often non‐diagnostic, early excisional biopsy is important. Rituximab‐based therapy represents a guideline‐supported alternative when anti‐IL‐6 treatment is unavailable or not feasible.

## 1 Introduction

Castleman disease (CD) is a rare lymphoproliferative disorder that can present with or without constitutional symptoms [1]. It occurs as either unicentric CD (UCD), involving a single lymph node, or multicentric CD (MCD), involving multiple lymph nodes and having a more aggressive course [2]. Idiopathic multicentric Castleman disease (iMCD), a rare CD subtype, is marked by systemic inflammation, generalized lymphadenopathy, and multiorgan involvement driven by dysregulated cytokine activity [3]. Diagnosis requires integrating clinical, laboratory, radiologic, and histopathological findings and excluding mimics [3]. The TAFRO (thrombocytopenia, anasarca, fever, reticulin fibrosis/renal dysfunction, organomegaly) variant of iMCD is usually rapidly progressive [4], but iMCD severity and progression can vary and may not fit classical subtypes [5]. We describe a case of iMCD‐TAFRO presenting with prolonged constitutional symptoms and generalized lymphadenopathy, ultimately diagnosed using comprehensive clinicopathological correlation.

## 2 Case History and Examination

A 21‐year‐old male presented to the OPD with a three‐year history of intermittent low‐grade fever and constitutional symptoms, a two‐year history of bilateral cervical lymphadenopathy, and a four‐week history of progressive abdominal distension. Fevers (up to 100°F) occurred in evening clusters without chills, rigors, or night sweats. At presentation, he had been experiencing febrile episodes for the preceding 4 weeks. There was associated anorexia and weight loss of approximately 15 kg (23% of his recorded body weight) over the previous 6 months. The cervical swellings were bilateral, painless, insidious in onset, and gradually progressive, without associated skin changes. The reports of a previous fine‐needle aspiration cytology (FNAC) were unavailable. Empirical anti‐tubercular therapy did not yield clinical improvement. Abdominal distension developed gradually over 4 weeks with dragging pain, early satiety, bilateral pedal edema without facial puffiness, and occasional frothy urine. Additionally, he complained of an intermittent dry cough associated with right‐sided pleuritic chest pain. There was no history of other systemic complaints, tuberculosis contact, recent travel, previous similar episodes, known comorbidities, substance use, or high‐risk sexual behavior. He occasionally took antipyretics, was not on any regular medications, and had no known drug allergies.

On examination, he was conscious, alert, and oriented to time, place, and person. He was febrile, with a recorded temperature of 100.1°F. His pulse rate was 104 beats/min and regular; blood pressure was 110/68 mmHg; respiratory rate was 16 breaths/min; and oxygen saturation was 98% on room air. General examination revealed pallor and generalized lymphadenopathy. Multiple non‐tender, firm‐to‐rubbery lymph nodes were palpable in the bilateral cervical and inguinal regions and the right axilla, with the largest dimension measuring approximately 2 cm in the cervical region, 2.5 cm in the right axilla, and 2–3 cm in the inguinal regions. Bilateral pitting pedal edema (2+) extending up to the knees was also observed. Abdominal examination revealed marked distension. On palpation, hepatosplenomegaly was noted: The spleen was palpable 4–5 cm below the left costal margin and the liver 1–2 cm below the right costal margin, with an estimated liver span of 18 cm. There was no abdominal tenderness, guarding, or rebound tenderness. Percussion demonstrated shifting dullness, consistent with significant ascites. Respiratory examination revealed reduced breath sounds over the right infra‐scapular and basal regions, suggestive of a right‐sided pleural effusion. Examination of other systems was unremarkable.

## 3 Methods

### 3.1 Differential Diagnoses

The main differential diagnoses considered during hospitalization, along with the supporting and opposing clinical features for each, are summarized in Table 1.

**TABLE 1:** Differential diagnoses considered during the course of hospitalization.

Diagnosis	Points in favor	Points against
*Infectious etiologies* Disseminated tuberculosis with pleural and peritoneal involvement	Chronic history of intermittent low‐grade fever (100°F) with constitutional symptoms Evening rise in temperature Generalized lymphadenopathy Significant weight loss Pleural effusion Massive ascites	Lack of improvement in symptoms despite taking ATT for 9 months Low ADA levels in the ascitic fluid SAAG > 1.1 mg/dl Pleural and ascitic fluid analysis ruled out infective etiology
EBV lymphoproliferative disorders	Generalized lymphadenopathy Constitutional symptoms	Negative EBV serology Histopathology did not support an EBV‐associated lymphoproliferative disorder
HIV	Chronic history of constitutional symptoms Generalized lymphadenopathy Significant weight loss	Lack of high‐risk behaviors (IV drug use/unsafe sex practices) Negative serology
*Malignant etiologies* Hodgkin and Non‐Hodgkin's lymphoma with pleural and peritoneal involvement	Chronic history of intermittent low‐grade fever (100°F) with constitutional symptoms Generalized lymphadenopathy Significant weight loss Pleural effusion Massive ascites Elevated LDH levels	Ruled out histopathologically Ascitic and pleural fluid examination did not demonstrate findings of malignancy
Leukemia (with serosal infiltration)	Chronic history of intermittent low‐grade fever (100°F) with constitutional symptoms Significant weight loss Pleural effusion Massive ascites Elevated LDH levels Anemia and Thrombocytopenia	Peripheral smear and bone marrow findings did not suggest leukemia
*Autoimmune etiologies* SLE	Multisystemic involvement ANA positivity Pancytopenia	Did not fulfill the 2019 EULAR/ACR classification criteria for SLE despite ANA positivity
Idiopathic multicentric Castleman disease (TAFRO subtype)	Generalized lymphadenopathy Fever and constitutional symptoms Thrombocytopenia Anasarca (massive ascites and pleural effusions) Elevated inflammatory markers and IL‐6 Renal involvement (MPGN) Histopathology demonstrating features consistent with Castleman disease	Rare disease with significant clinical overlap with infections, malignancy, and autoimmune disorders, making diagnosis challenging

Abbreviations: AATT, anti‐tubercular therapy; ADA, adenosine deaminase; ANA, antinuclear antibody; EBV, Epstein‐Barr virus; HIV, human immunodeficiency virus; IL‐6, interleukin‐6; IV, intravenous; LDH, lactate dehydrogenase; MPGN, membranoproliferative glomerulonephritis; SAAG, serum–ascites albumin gradient; SLE, systemic lupus erythematosus; TAFRO, thrombocytopenia, anasarca, fever, renal failure/reticulin fibrosis, organomegaly.

### 3.2 Investigations

Initial laboratory and imaging results are summarized in Tables 2 and 3. FNAC of the cervical lymph node was also planned but proved inconclusive, prompting excisional biopsy for definitive diagnosis. Ongoing fever, generalized lymphadenopathy, pancytopenia, proteinuria, and multisystem involvement led to an extensive immunological, infectious, inflammatory, and cardiac evaluation along with a contrast‐enhanced CT (CECT) scan of the abdomen, upper gastrointestinal endoscopy (UGIE), transient elastography (FibroScan), and bone marrow biopsy. Two units of packed RBCs were transfused for anemia, and supportive care with clinical monitoring continued.

**TABLE 2:** Initial laboratory investigations.

Investigation	Key parameters	Findings	Reference range for males	Impression
CBC	Hemoglobin, TLC, Platelets, Peripheral smear	Hb 7.8 g/dL TLC 3230/μL Platelets 99,000/μL Occasional atypical lymphocytes	14–18 g/dL 4,000–11,000/μL 150–400 × 10^3^/μL	Pancytopenia
2 LFT	Albumin, ALP, ALT, AST, Bilirubin	Albumin 3.0 g/dL ALP 280 U/L ALT 13 U/L AST 23 U/L Bilirubin 0.4 mg/dL	3.5–5 g/dL 45–115 U/L 7–55 U/L 8–48 U/L 0.1–1.2 mg/dL	Hypoalbuminemia with elevated ALP
3 KFT and Electrolytes	Urea, Creatinine, Sodium, Potassium	Urea 20 mg/dL Creatinine 0.8 mg/dL Na 138 mmol/L K 4.2 mmol/L	15–40 mg/dL 0.7–1.2 mg/dL 135–145 mmol/L 3.5–5 mmol/L	Within normal limits
4 Ascitic Fluid Analysis	Appearance, SAAG, Protein, Cell count	Clear straw‐colored SAAG 1.3 g/dL Sugar 82 mg/dL Protein 4.2 g/dL 90 cells/μL (mononuclear) ADA 13.2 IU/L	SAAG ≥ 1.1 g/dL: Portal hypertension Protein > 2.5 g/dL: High‐protein ascites ADA < 40 IU/L; Cells < 250/μL	High SAAG, high‐protein ascites; no evidence of SBP or tuberculosis; cytology negative
5 Pleural Fluid Analysis	TLC, DLC, LDH, ADA	TLC 200 cells/μL DLC (Lymphocytes: 70%) LDH 203 U/L ADA 12 U/L	Interpretation based on Light's criteria	Findings suggest transudative effusion; cytology negative

Abbreviations: ADA, adenosine deaminase; ALP, alkaline phosphatase; ALT, alanine aminotransferase; AST, aspartate aminotransferase; CBC, complete blood count; DLC, differential leukocyte count; g/dL, grams per deciliter; Hb, hemoglobin; KFT, kidney function test; LDH, lactate dehydrogenase; LFT, liver function test; mg/dL, milligrams per deciliter; SAAG, serum–ascites albumin gradient; TLC, total leukocyte count; U/L, units per liter; ×10^3^/μL, thousand cells per microliter.

**TABLE 3:** Initial imaging studies.

Modality	Key findings	Impression
Ultrasound (Hepato‐Biliary System)	Hepatosplenomegaly, portal hypertension, ascites	Hepato‐splenomegaly with gross ascites Dilated hepatic veins
2 Ultrasound Neck	Bilateral cervical lymphadenopathy	Multiple enlarged nodes, largest 2.1 × 1.3 cm in measurement, preserved fatty hilum
3 Chest X‐ray	Bilateral hilar prominence with pleural effusion	Right‐sided pleural effusion

Tables 4 and 5 summarize the results of the additional, relevant lab and imaging findings.

**TABLE 4:** Lab investigations performed in the first week of hospitalization.

Immunology & Infection	C3	159 mg/dL	90–180 mg/dL
	C4	37.1 mg/dL	10–40 mg/dL
	Kappa/Lambda Ratio	1.16/1.50	0.26–1.65
	IgG	471 mg/dL	700–1,600 mg/dL
	IgM	171 mg/dL	40–230 mg/dL
	IgA	345 mg/dL	70–400 mg/dL
	ANA Profile	Negative	Negative
	ANCA	Negative	Negative
	Serum Immunofixation	Negative	Negative
	Tuberculosis ELISA	Negative	Negative
	RK39	Negative	Negative
Urine Analysis	Spot Urine Albumin	120.6 mg/g	< 30 mg/g
	Urine Creatinine	88 mg/dL	Variable
	24‐h Urine Protein	1370.45 mg/day	< 150 mg/day
	Urine pH	Acidic	4.5–8.0
	Urine Albumin	2+	Negative
	Sugar	Nil	Negative
	Pus Cells	Nil	0–5/HPF
	RBCs	Nil	0–2/HPF
Inflammatory & Cardiac Markers	CRP	43 mg/L	< 5 mg/L
	Procalcitonin	0.87 ng/mL	< 0.1 ng/mL
	BNP	845 pg/mL	< 100 pg/mL

Abbreviations: ANA, antinuclear antibody; ANCA, anti‐neutrophil cytoplasmic antibody; BNP, B‐type natriuretic peptide; C3, complement component 3; C4, complement component 4; CRP, C‐reactive protein; ELISA, enzyme‐linked immunosorbent assay; HPF, high‐power field; IgA, immunoglobulin A; IgG, immunoglobulin G; IgM, immunoglobulin M; RBCs, red blood cells; RK39, recombinant kinesin‐39 antigen test.

**TABLE 5:** Additional imaging studies performed for the patient.

Investigations	Findings
Upper gastro‐intestinal endoscopy	Mild portal hypertensive gastropathy in fundus and body; rest of mucosa normal; No esophageal or gastric varices
FNAC	Inconclusive; Excision biopsy planned
2D Echocardiography	EF ~60%; Normal study
CECT Abdomen	Gross free ascites Hepatomegaly (~17.9 cm, Beaver's tail appearance, no focal lesions) Splenomegaly (15.2 cm) Prominent portal vein (14 mm, patent spleno‐portal axis) Distended gallbladder with mild pericholecystic fluid Mild perinephric fat stranding with thickened anterior/lateral conal fascia Multiple homogeneously enhancing subcentimetric mesenteric lymph nodes (largest ~9.6 mm)
FibroScan	15.5 kPa/CAP 25.3 dB/m

Abbreviations: CAP, controlled attenuation parameter; CECT, contrast‐enhanced computed tomography; dB/m, decibels per meter; EF, ejection fraction; FNAC, fine‐needle aspiration cytology; kPa, kilopascals.

Second‐week lymph node excision biopsy revealed features consistent with CD (atretic follicles, interfollicular vascular proliferation, hyalinized vessels, concentric mantle zone; Figures 1, 2, 3). Further testing: Serum interleukin‐6 (IL‐6) levels, Epstein‐Barr virus (EBV) serology, human herpesvirus‐8 (HHV‐8) serology, LANA‐1 immunohistochemistry for the lymph node biopsy, and serum protein electrophoresis (SPEP) were performed to characterize the disease and exclude secondary causes. Persistent proteinuria led to a renal biopsy for glomerular pathology. Initial autoimmune screening was negative, but due to ongoing multisystemic involvement, an expanded immunological workup was repeated.

**FIGURE 1:**
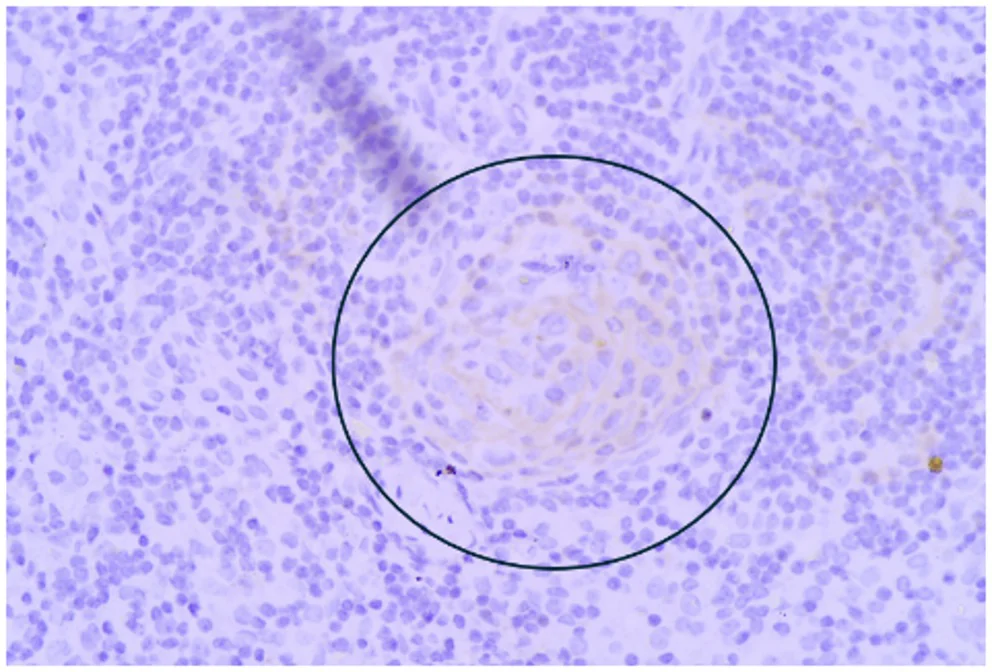
Level 4 cervical lymph node biopsy demonstrating an atretic follicle (40×).

**FIGURE 2:**
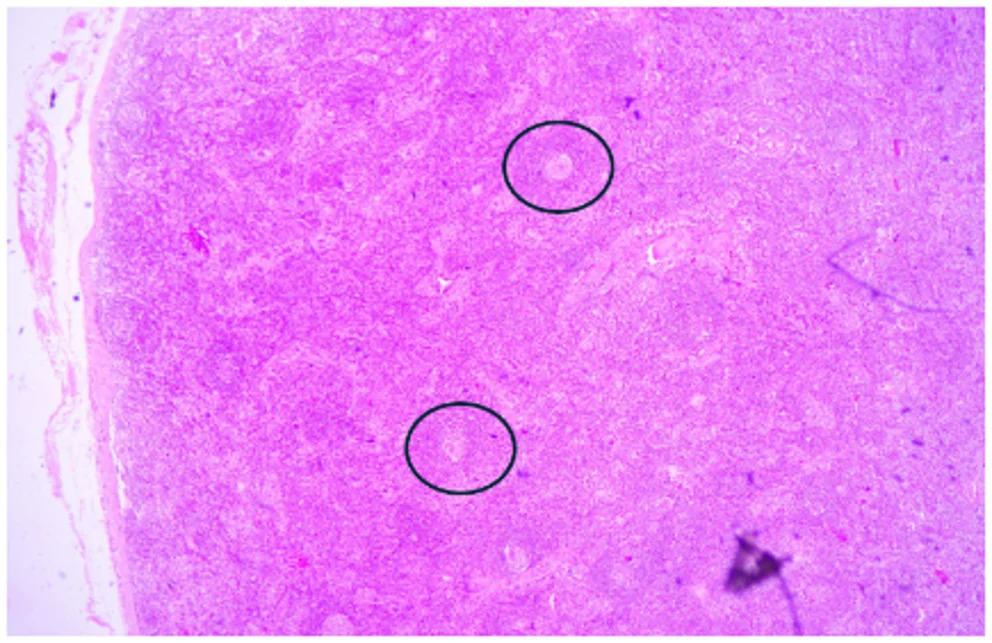
Level 4 cervical lymph node biopsy demonstrating interfollicular vascular proliferation (2×).

**FIGURE 3:**
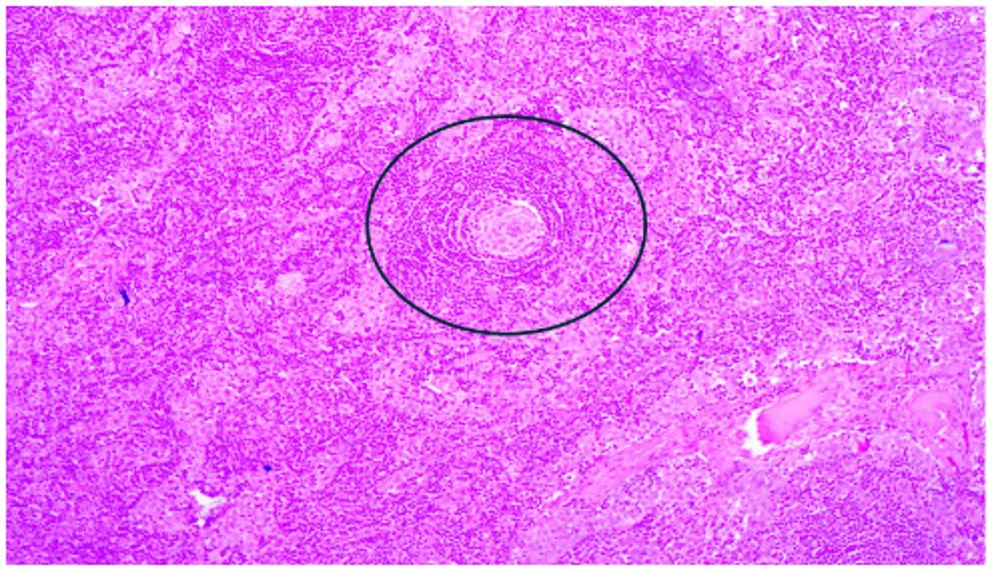
Level 4 cervical lymph node biopsy demonstrating a hyalinized vessel in the center giving an onion‐skin appearance (10×).

By the third week, the patient improved clinically with reduced fever. Renal biopsy revealed features consistent with membranoproliferative glomerulonephritis (MPGN), raising the possibility of secondary renal involvement and explaining the proteinuria. However, the etiology of the portal hypertension and ascites remained unclear; in view of this, a liver biopsy was performed, which was nearly normal. Generalized lymphadenopathy, constitutional symptoms, hepatosplenomegaly, elevated inflammatory markers, and characteristic histopathological findings led to the diagnosis of MCD, following which rituximab therapy was initiated.

The additional investigations undertaken to characterize the disease subtype and exclude secondary causes revealed elevated serum IL‐6 levels (24 pg/mL; reference < 7 pg/mL), negative EBV and HHV‐8 serology, and negative LANA‐1 staining of the excised lymph node, supporting a diagnosis of idiopathic MCD. Bone marrow examination demonstrated reactive erythroid hyperplasia without evidence of marrow infiltration, hematological malignancy, or reticulin fibrosis. Repeat immunological workup showed seroconversion with positive ANA and anti‐dsDNA antibodies, along with reactive p‐ANCA, an indeterminate c‐ANCA result, and negative anti‐CCP antibodies. The SPEP also showed no significant findings.

Characteristic histopathological features, the presence of enlarged lymph nodes in at least two lymph node stations, elevated CRP levels, anemia, thrombocytopenia, renal dysfunction, proteinuria, hypoalbuminemia, constitutional symptoms, hepatosplenomegaly, and ascites led to a diagnosis of iMCD. Negative LANA‐1 staining, combined with thrombocytopenia, anasarca, elevated CRP, renal dysfunction, organomegaly, and biopsy‐confirmed renal involvement, supported the final diagnosis of iMCD with TAFRO syndrome, MPGN, and suspected secondary Budd–Chiari syndrome.

### 3.3 Treatment

The patient was started on systemic corticosteroid therapy at a dose of 1 mg/kg/day with improvement in fever and inflammatory manifestations. Subsequently, intravenous rituximab 500 mg weekly was administered for 4 weeks.

### 3.4 Outcome and Follow‐Up

He demonstrated a favorable clinical response. Fever subsided completely following initiation of therapy, with progressive improvement in constitutional symptoms, appetite, and weight. On follow‐up, there was complete resolution of ascites, pleural effusion, and generalized lymphadenopathy. Functional status improved significantly, allowing the patient to resume his routine daily activities and return to work. Prednisolone was gradually tapered over 6 months and maintained at 5 mg on alternate days. The patient was advised regular follow‐up with clinical assessment and interval CECT of the neck, chest, and abdomen to monitor treatment response and detect relapse.

The chronological sequence of the patient's clinical presentation, diagnostic evaluation, and treatment course is summarized in Table 6.

**TABLE 6:** Chronological sequence of the patient's clinical presentation, diagnostic evaluation, and treatment course.

Timeframe	Clinical Events, Diagnostics, and Management
3 Years Prior to Admission	Onset of intermittent low‐grade fever (up to 100°F) occurring in clusters, accompanied by constitutional symptoms, loss of appetite, and progressive weight loss.
2 Years Prior to Admission	Development of bilateral, painless cervical lymphadenopathy, insidious in onset.
9 Months Prior to Admission	Initial evaluation at a local healthcare facility, including FNAC (reports unavailable). Empirical ATT was initiated and continued for 9 months without clinical improvement.
4 Weeks Prior to Admission	Development of progressive abdominal distension, early satiety, right‐sided pleuritic chest pain, dry cough, frothy urine, and bilateral pedal edema.
Week 1 of Hospitalization	Admission and Baseline Evaluation: Physical examination revealed generalized lymphadenopathy, hepatosplenomegaly, ascites, and pleural effusion. Laboratory investigations demonstrated pancytopenia, hypoalbuminemia, elevated alkaline phosphatase with normal transaminases and elevated C‐reactive protein. Ascitic fluid analysis showed high SAAG, high‐protein ascites. Initial autoimmune screening was negative. CECT of the abdomen confirmed hepatosplenomegaly and ascites. An inconclusive FNAC prompted planning an excisional lymph node biopsy. Supportive care was administered.
Week 2 of Hospitalization	Excisional Biopsy and Corticosteroid Initiation: Excisional cervical lymph node biopsy demonstrated atretic lymphoid follicles, interfollicular vascular proliferation, and concentric “onion‐skin” mantle zones, consistent with Castleman disease. Oral prednisolone (1 mg/kg/day) was initiated. Additional investigations, including IL‐6, viral serologies, autoimmune panel, renal and liver biopsies, and LANA‐1 staining of the lymph node biopsy were undertaken. Initial autoimmune screening was negative, but due to ongoing multisystemic involvement, an expanded immunological workup was repeated.
Week 3 of Hospitalization	Definitive Diagnosis and Targeted Immunotherapy: Renal biopsy revealed membranoproliferative glomerulonephritis. Additional investigations revealed elevated serum IL‐6, negative HHV‐8 and EBV serologies and negative LANA‐1 staining of the biopsy. Liver biopsy revealed a normal liver morphology. Repeat immunological workup showed seroconversion with positive ANA and anti‐dsDNA antibodies, along with reactive p‐ANCA, an indeterminate c‐ANCA result, and negative anti‐CCP antibodies. Characteristic histopathological features, the presence of enlarged lymph nodes in at least two lymph node stations, elevated CRP levels, anemia, thrombocytopenia, renal dysfunction, proteinuria, hypoalbuminemia, constitutional symptoms, hepatosplenomegaly, and ascites led to a diagnosis of iMCD. The negative LANA‐1 staining, combined with thrombocytopenia, anasarca, elevated CRP, renal dysfunction, organomegaly, and biopsy‐confirmed renal involvement, supported the final diagnosis of iMCD with TAFRO syndrome, MPGN, and suspected secondary Budd–Chiari syndrome. The patient received rituximab 500 mg weekly for 4 weeks and continued oral prednisolone 40 mg daily.
6‐Month Follow‐Up	Complete clinical and radiological resolution of fever, ascites, pleural effusion, and generalized lymphadenopathy. The patient achieved full functional recovery and returned to work. Prednisolone was successfully tapered to 5 mg on alternate days.

Abbreviations: ALP, alkaline phosphatase; ATT, antitubercular therapy; CECT, contrast‐enhanced CT; EBV, Epstein‐Barr virus; FNAC, fine‐needle aspiration cytology; HHV‐8, human herpesvirus 8; IL‐6, interleukin 6; LANA‐1, latency‐associated nuclear antigen 1; MPGN, membranoproliferative glomerulonephritis; SAAG, serum–ascites albumin gradient; TAFRO, thrombocytopenia, anasarca, fever, renal dysfunction/reticulin fibrosis, organomegaly.

## 4 Discussion

CD encompasses at least four disorders that share common histopathological features but differ greatly in their causes, manifestations, treatments, and outcomes. Recently, the Castleman Disease Collaborative Network (CDCN) introduced a classification system that expands beyond the traditional unicentric versus multicentric dichotomy to recognize oligocentric CD (OligoCD), an intermediate phenotype characterized by regional lymphadenopathy involving 2–4 lymph node stations with mild systemic symptoms that falls between unicentric and multicentric disease on the clinical spectrum [6]. Within multicentric CD, the CDCN classification subdivides MCD based on underlying causes—namely, HHV‐8–associated MCD (HHV‐8‐MCD), which is further divided by HIV serostatus; POEMS (polyneuropathy, organomegaly, endocrinopathy, M‐protein, skin changes) syndrome–related MCD (POEMS‐MCD); and idiopathic MCD (iMCD). The iMCD category is further divided into subtypes: iMCD‐TAFRO (characterized by thrombocytopenia, anasarca, fever, reticulin fibrosis/renal dysfunction, and organomegaly); iMCD–not otherwise specified (NOS); and idiopathic plasmacytic lymphadenopathy (iMCD‐IPL), a distinct subtype marked by thrombocytosis, polyclonal hypergammaglobulinemia, and less severe fluid accumulation that typically follows a more indolent course [7].

Diagnosis of all four subtypes of CD begins with an excised lymph node biopsy showing the typical histopathological features classic for CD [8].

While characteristic histopathological features are vital for diagnosing CD, they are not sufficient on their own. The diagnosis involves combining histopathological, clinical, laboratory, and radiological data. Figure 4 [3] outlines the inclusion criteria for iMCD. It is equally important to rule out any other etiology that might be the cause of the patient's multisystemic presentation, including infection‐related disorders (HHV‐8, EBV‐associated lymphoproliferative disorder, any other infection that might cause inflammatory adenopathy), autoimmune or inflammatory diseases (SLE, rheumatoid arthritis, adult‐onset Still's disease, juvenile idiopathic arthritis, autoimmune lymphoproliferative syndrome), and malignant lymphoproliferative disorders [3].

**FIGURE 4:**
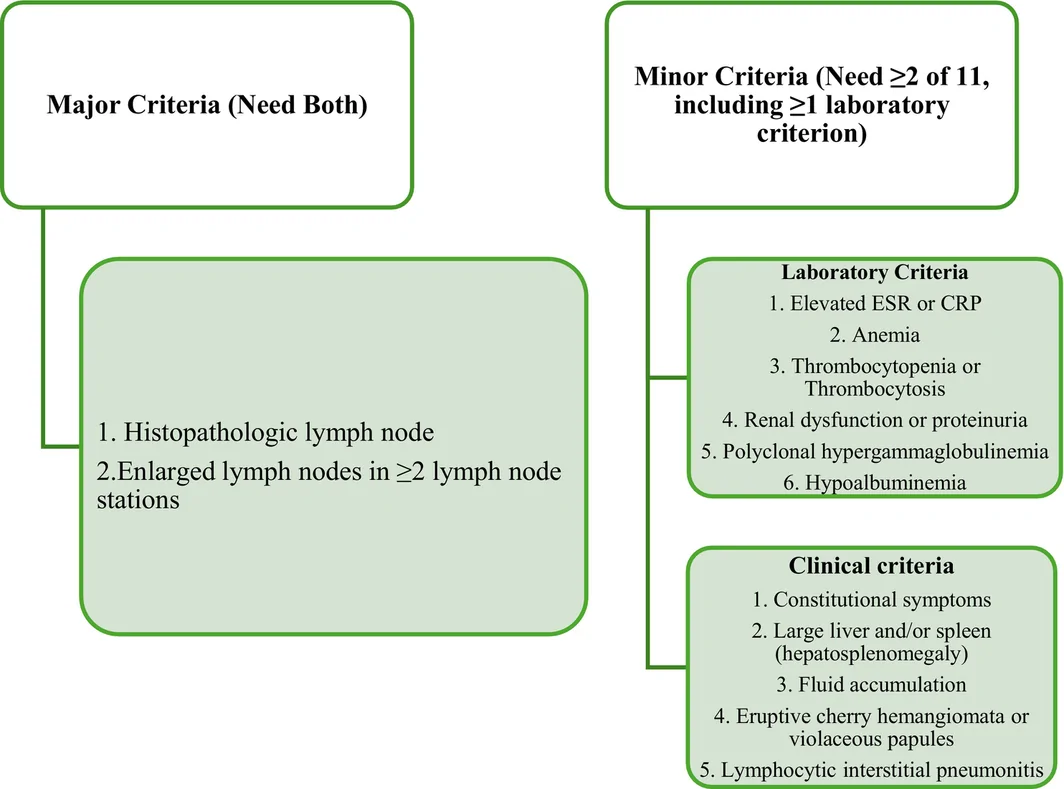
Inclusion criteria for iMCD. Redrawn by the authors based on data from Fajgenbaum et al. [3]. ESR: Erythrocyte sedimentation rate; CRP: C‐reactive protein.

Our patient fulfilled the diagnostic criteria for iMCD. He met both mandatory major criteria, namely characteristic lymph node histopathology and lymphadenopathy involving multiple lymph node stations. In addition, the patient fulfilled several minor criteria, including elevated inflammatory markers (CRP), anemia, thrombocytopenia, hypoalbuminemia, constitutional symptoms (persistent fever and weight loss), hepatosplenomegaly, fluid accumulation in the form of ascites and pleural effusion, and renal involvement manifesting as significant proteinuria with biopsy‐proven MPGN. A comprehensive diagnostic workup was undertaken to rule out other mimickers.

The TAFRO subtype of iMCD is characterized by thrombocytopenia, anasarca, fever, reticulin fibrosis or renal dysfunction, and organomegaly. Table 7 [9] below describes the diagnostic criteria for iMCD‐TAFRO.

**TABLE 7:** Diagnostic criteria for iMCD‐TAFRO.

Category	Criteria
Histopathological criteria (all required)	Typical lymph node pathology (atrophic germinal centers with enlarged endothelial cell nuclei, proliferation of endothelial venules, and small numbers of mature plasma cells)
	Negative LANA‐1 for HHV‐8
Major criteria (≥ 3 of 5 required)	Thrombocytopenia (< 100,000/μL)
	Anasarca (pleural effusions and/or ascites on CT)
	Fever (> 38°C) or CRP ≥ 2.0 mg/dL
	Reticulin fibrosis or Renal dysfunction
	Organomegaly
Minor criteria (≥ 1 required)	Hyperplasia/Normoplasia of megakaryocytes
	Elevated alkaline phosphatase without markedly elevated transaminases

*Note:* Redrawn by the authors based on the data from Iwaki et al. [9].

Abbreviations: HHV‐8: human herpesvirus‐8; LANA‐1: latency‐associated nuclear antigen‐1.

Our patient met the diagnostic criteria for iMCD‐TAFRO, including histopathological criteria; thrombocytopenia; anasarca (massive ascites and pleural effusion); CRP ≥ 2.0 mg/dL (the patient had CRP levels of 4.3 mg/dL); renal involvement; organomegaly; and elevated alkaline phosphatase levels with relatively preserved transaminases.

The TAFRO subtype is generally regarded as the most clinically severe phenotype of i‐MCD. It commonly has a subacute‐to‐acute presentation, with rapid escalation of inflammation, capillary leak, cytopenias, and end‐organ dysfunction that may require hospitalization and intensive supportive measures [4]. In the present case, the term “indolent” should be interpreted with respect to the overall evolution of the underlying iMCD, rather than the tempo of the fully developed TAFRO phenotype. The prolonged constitutional phase preceded the onset of the characteristic TAFRO manifestations. The clinical TAFRO phenotype became apparent only when the patient developed the defining constellation of symptoms [4]. Accordingly, the principal observation in this case is not that the TAFRO phase itself remained indolent over a prolonged interval. Rather, it is that a prolonged constitutional illness compatible with underlying iMCD preceded the subsequent emergence of the clinically recognizable TAFRO phenotype, which complicated the early recognition of the disease [10]. Once the TAFRO phenotype emerged, the patient did not progress to the degree of life‐threatening organ failure or need for intensive supportive therapies that have been reported in many iMCD‐TAFRO cohorts. This is supported by a Western cohort study where patients with TAFRO did not have significantly worse two‐year overall survival than those with iMCD‐NOS, underscoring that the presence of TAFRO‐defining features does not invariably predict a uniformly catastrophic clinical course [11].

Renal involvement in iMCD‐TAFRO is well‐documented, with MPGN representing one of the two principal histopathologic patterns described in the literature, alongside thrombotic microangiopathy (TMA)‐like glomerulopathy. The MPGN pattern in TAFRO is thought to represent chronic TMA rather than primary immune‐complex‐mediated disease. Nagayama et al. [12] proposed that TMA appears in the acute phase with TMA‐like histology, while chronic phase biopsies demonstrate MPGN‐like changes. This temporal evolution suggests that the endothelial injury and mesangiolysis characteristic of TAFRO‐related TMA may organize into MPGN‐pattern injury over time. The IL‐6 vascular endothelial growth factor (VEGF) axis is implicated in the pathogenesis of TAFRO renal disease. Elevated serum VEGF and IL‐6 levels are frequently observed, with VEGF‐induced glomerular endothelial injury hypothesized as a central mechanism. This cytokine‐driven microangiopathy may explain why MPGN in TAFRO often lacks the immune deposits typical of primary immune‐complex MPGN [12].

This case also highlights the diagnostic challenges posed by iMCD, particularly in tuberculosis‐endemic regions. Chronic fever, weight loss, generalized lymphadenopathy, and elevated inflammatory markers commonly raise suspicion for more common diagnoses like tuberculosis or lymphoma, often delaying consideration of rarer disorders such as Castleman disease. Furthermore, fine‐needle aspiration cytology was non‐diagnostic in our patient, emphasizing the limited utility of cytology in such cases and reinforcing the importance of excisional lymph node biopsy as the diagnostic gold standard.

A striking feature of this case was the extent of systemic involvement. This multisystemic involvement reflects the systemic inflammatory nature of the disease, largely driven by dysregulated cytokine activity. The literature states that IL‐6 is a critical disease driver in some patients [13, 14]. However, it is not universally elevated in iMCD, and approximately one‐half of patients do not respond to IL‐6 inhibition. Alternative cytokines and signaling pathways are likely involved in these cases. Recently, a key role for activated T cells and mammalian target of rapamycin pathway activation has been observed, which has led to the use of mammalian target of rapamycin inhibitors with early success. The cause of the increased IL‐6 and other cytokines in iMCD is unknown [15].

An additional diagnostic dilemma in this case was the presence of transient autoimmune serological abnormalities, including positive ANA, anti‐dsDNA, and p‐ANCA antibodies. The presence of these in a patient with iMCD‐TAFRO reflects shared pathophysiological mechanisms between iMCD and systemic autoimmune diseases, particularly involving Type I interferon signaling, IL‐6‐driven B‐cell dysregulation, and endothelial activation [16]. Although current diagnostic criteria require the exclusion of autoimmune diseases that can mimic iMCD, emerging evidence indicates that these autoantibodies may coexist with iMCD‐TAFRO without meeting criteria for a distinct autoimmune diagnosis, reflecting a spectrum of immune dysregulation rather than mutually exclusive conditions [17].

For all patients with iMCD, regardless of disease severity, the algorithm starts with anti–IL‐6–directed therapy. Siltuximab, an anti–IL‐6 antibody, is the only US Food and Drug Administration (FDA)‐approved treatment of iMCD [18]. Data from the use of tocilizumab, an anti–IL‐6 receptor antibody approved for iMCD in Japan based on a single‐arm open‐label study, suggest that relapses occur upon cessation of therapy [11]. Overall, both therapies are well tolerated with the most common side effects being hyperlipidemia, mild thrombocytopenia, and pruritus. IL‐6 levels become uninterpretable while patients are on active anti–IL‐6 therapy and cannot be used to monitor response for 18 to 24 months following the last dose of siltuximab. Moreover, lymph node responses are often delayed in patients receiving anti–IL‐6 antibody monotherapy compared with chemotherapy. The most reliable measures are hemoglobin, ESR, CRP, albumin, and clinical symptomology [19].

For patients with non‐severe iMCD who do not respond to IL‐6 blockade, many therapies can be tried, including chemotherapy and immunomodulatory therapies, and it is difficult to recommend one over another. The general rule, however, is to avoid cytotoxic chemotherapy as long as the patient does not have severe disease with progressive organ dysfunction [19].

Because iMCD‐TAFRO is a relatively newly recognized subtype, there is even less information about the best therapies for these patients. They can respond to anti‐IL‐6 therapies, calcineurin inhibitors like tacrolimus, and other immune‐modulatory drugs like sirolimus, but data are mostly anecdotal [20, 21, 22].

Siltuximab and tocilizumab are not universally available due to significant geographic and economic barriers limiting access in many regions, including India. In this context, we initiated rituximab in combination with corticosteroids. Current guidelines explicitly permit its use when IL‐6–targeted agents are unavailable or not feasible. The 2018 CDCN (Castleman Disease Collaborative Network) and 2026 NCCN (National Comprehensive Cancer Network) guidelines list rituximab as an option for patients with severe iMCD who are “not candidates for combination chemotherapy” or when anti‐IL‐6 therapy is inaccessible [23]. Real‐world data from the ACCELERATE registry demonstrate that 26.9% of patients treated with rituximab with/without corticosteroids achieved response, supporting its efficacy [10]. Furthermore, emerging evidence supports B‐cell–mediated pathogenesis in a subset of iMCD‐TAFRO, providing mechanistic rationale for rituximab beyond simple IL‐6 blockade. A systematic review of 212 TAFRO cases found that rituximab was significantly more effective in anti‐SSA antibody–positive patients (88.9% response) than in antibody‐negative patients (16.7%), suggesting that B‐cell dysregulation may drive disease in specific subgroups [17]. Our patient's positive autoantibody profile, including ANA, anti‐dsDNA, and p‐ANCA, may reflect underlying immune dysregulation; however, these findings should not be interpreted as evidence that autoantibody positivity predicts response to rituximab, particularly in an individual case. Resource‐limited settings necessitate pragmatic deviation from guideline‐preferred IL‐6 blockade. However, as a single‐case observation, it cannot establish the efficacy of rituximab or its comparative effectiveness relative to anti‐IL‐6 therapy. Further prospective studies are needed to define the optimal role and sequencing of rituximab in iMCD‐TAFRO.

iMCD, particularly the TAFRO subtype, remains a rare and underreported entity, with most of the available literature limited to small case series and isolated case reports [3]. The rarity is further compounded by diagnostic challenges arising from its clinical overlap with other common etiologies, often leading to under‐recognition in routine practice [24]. Although cases have been increasingly reported worldwide, published data from India remain sparse, with only a limited number of documented cases. In this context, the present report contributes to the existing knowledge by describing a rare presentation of Castleman disease from India, thereby adding to the geographically diverse but still limited pool of reported iMCD‐TAFRO cases [25].

In conclusion, this case underscores the diagnostic challenge posed by iMCD‐TAFRO, particularly in regions with a high burden of tuberculosis where diagnostic overlap can lead to significant delays. The transient autoantibodies and concurrent renal pathology further highlight the multisystem nature of this syndrome. Maintaining a high index of suspicion and timely excisional lymph node biopsy are essential when evaluating unresolved generalized lymphadenopathy. Furthermore, when standard first‐line anti–interleukin‐6 therapy is logistically or financially inaccessible, B‐cell depletion using rituximab‐based therapy represents a guideline‐supported alternative. While our patient improved clinically after rituximab, this single case does not establish its efficacy or superiority over anti‐IL‐6 therapy. Further evidence is needed to better define rituximab's role in such settings.

## Author Contributions

**Divita Rohatgi:** writing – original draft, writing – review and editing, conceptualization, data curation, methodology. **Annesha Chakraborty:** formal analysis, supervision, validation, visualization. **Sourav Kumar:** supervision, validation, visualization. **Anurag Rohatgi:** supervision, formal analysis.

## Funding

The authors have nothing to report.

## Consent

Written informed consent to report the case and for the patient information and images to be published was provided by the patient.

## Data Availability

All relevant data supporting the findings of this case report are included within the manuscript.
